# *NKX2.1*-Related Disorders: a novel mutation with mild clinical presentation

**DOI:** 10.1186/s13052-015-0150-6

**Published:** 2015-06-24

**Authors:** Sara Monti, Annalisa Nicoletti, Antonella Cantasano, Heiko Krude, Alessandra Cassio

**Affiliations:** Department of Medical and Surgical Sciences, Pediatric Unit, University of Bologna, Bologna, Italy; Institute for Experimental Pediatric Endocrinology, Charité University Medicine, Berlin, Germany; Policlinico S.Orsola- Malpighi, U.O. Pediatria, Via Massarenti 9, 40138 Bologna, BO Italy

**Keywords:** *NKX2.1*-related disorders, Syndromic hypothyroidism, Neonatal screening, Choreoathetosis

## Abstract

**Background:**

A highly variable phenotype characterized by thyroid, respiratory and neurological defects has been reported in an already established group of disorders namely *NKX2.1*-related disorders. We describe here the case of an infant with a novel mutation of the *NKX2.1* gene characterized by mild clinical presentation.

Aim of the study was to elucidate the genotype-phenotype correlation in our patient.

**Methods:**

We performed genetic analysis of the *NKX2.1* gene in an infant with no neonatal respiratory distress and near-normal results at neonatal screening test for congenital hypothyroidism, choreoathetosis, ataxia and delayed independent walking.

**Results:**

A novel mutation of the *NKX2.1* gene has been identified, that is responsible for a mild framework of congenital hypothyroidism and neurological symptoms.

**Conclusions:**

The frequency of congenital hypothyroidism cases associated with *NKX2.1* mutations is expected to be higher in a subgroup of patients, selected according to the neurological presentation. In these patients the analysis of *NKX2.1* mutational status is recommended.

## Background

The management of the classical form of congenital hypothyroidism (CH) has changed thanks to the introduction of neonatal screening programs, which allow early diagnosis and replacement therapy. Over the last few years the progressive reduction of TSH-cutoff at neonatal screening and the advancements in the molecular analysis led to the definition of novel syndromic forms of CH, that are associated with mild thyroid deficiency [1].

Mutations of the *NKX2.1* gene, previously known as *TTF1* (thyroid transcription factor 1, OMIM*600635), mapping on chromosome 14q13, have been identified and associated with a syndromic form of CH (OMIM*610978) [2], initially named brain-lung-thyroid syndrome [3] due to the broad phenotypic spectrum including a variable combination of lung, thyroid and neurological defects. Recently, in light of the varied manifestations of heterozygous mutations of the *NKX2.1* gene, the disorders have been referred to as *NKX2.1*-related disorders [4]. These disorders range from benign hereditary chorea (BHC), the hallmark of *NKX2.1*-related disorders, to choreoathetosis, congenital hypothyroidism and neonatal respiratory distress [5–13].

So far, ninety-six mutations of the *NKX2.1* gene have been reported , 70 of which are point mutations and 26 large deletions, often spanning the entire gene (HGMD Professional 2014.2).

The *NKX2.1* gene has three exons and encodes for a member of the NK-2 family of transcription factors [14] that mediates thyroid-specific genes transcription. During embryogenesis the gene is expressed in the thyroid gland, in the lung and in the forebrain, especially in the hypothalamic area, infundibulum and basal ganglia region [15–17].

We identified a novel mutation of *NKX2.1* in an infant showing neurological symptoms associated with near-normal results at CH screening test, negative history of neonatal respiratory distress and recurrent pulmonary diseases.

## Case presentation

Child of non consanguineous parents, the patient was born at 41 weeks of normal pregnancy, with normal birth weight (3965 g). The family history reported two cases of walking delay in the paternal line and several cases of autoimmune thyroid diseases in the maternal line. Both parents showed normal thyroid function. Neonatal adaptation was normal (APGAR 1° 9, 5° 10), with no respiratory distress at birth. At neonatal screening, TSH spot value was equal to the cut-off level (10 mUI/L). The test was repeated on blood spot and TSH levels became normal (4.4 mUI/L), thus no further analysis was performed. In the first months of life, the infant showed a progressive impairment in neurological development, characterized by choreoathetosic movements, disordered motility in the lower limbs, reduced motility of the upper limbs, axial hypotonia and development delay. At 8 months of age the analysis revealed a mild hypertireotropinemia with normal fT4 values. Thyroid ultrasound showed a normal in situ gland. Electroencephalography and brain MRI were normal at 12 months of age. Due to the progressive increase of TSH levels, the infant started the replacement therapy with L- Thyroxin at 13 months. Growth parameters and pulse rate were recorded, electrocardiography was performed and blood samples were taken in order to determine TSH and free T4 serum levels. Biochemical and clinical features measured at follow-up are reported in Table 1.

**Table 1:** Patient’s laboratoristic and neurological features before treatment with L-thyroxine

	4th day	10th day	8th month	13th month
TSH spot value (mUI/L)	10	4.4		
TSH serum value (Normal range in our laboratory: 0.5-5 mUI/L)			8.8	13.8
fT4 serum value (Normal range in our laboratory: 10.5–22 pmol/L)			14.8	13.4
Choreoathetosis	-	-	+ −	++

Serum free T4 and TSH levels were measured using a commercial chemiluminescent assay (Bayer, Fenwald, Germany). The clinical manifestation of the patient resembled defects that have been previously associated with mutations of the *NKX2.1* gene. Therefore, DNA sequencing analysis was performed after obtaining informed consent. Genomic DNA was extracted from peripheral blood leukocytes of the child and his parents and the *NKX2.1* gene coding region, comprising exon 1–3 of isoform 1, as well as the exon-intron boundaries were amplified by PCR and directly sequenced by Sanger sequencing.

Two point mutations were identified, a heterozygous substitution, NM_001079668:c.390C > G:p.(Tyr130Term), resulting in a nonsense mutation and a single base exchange in intron 2, NM_ 001079668:c.463 + 41C > T, not reported in the SNP database (NCBI).

**Fig. 1:**
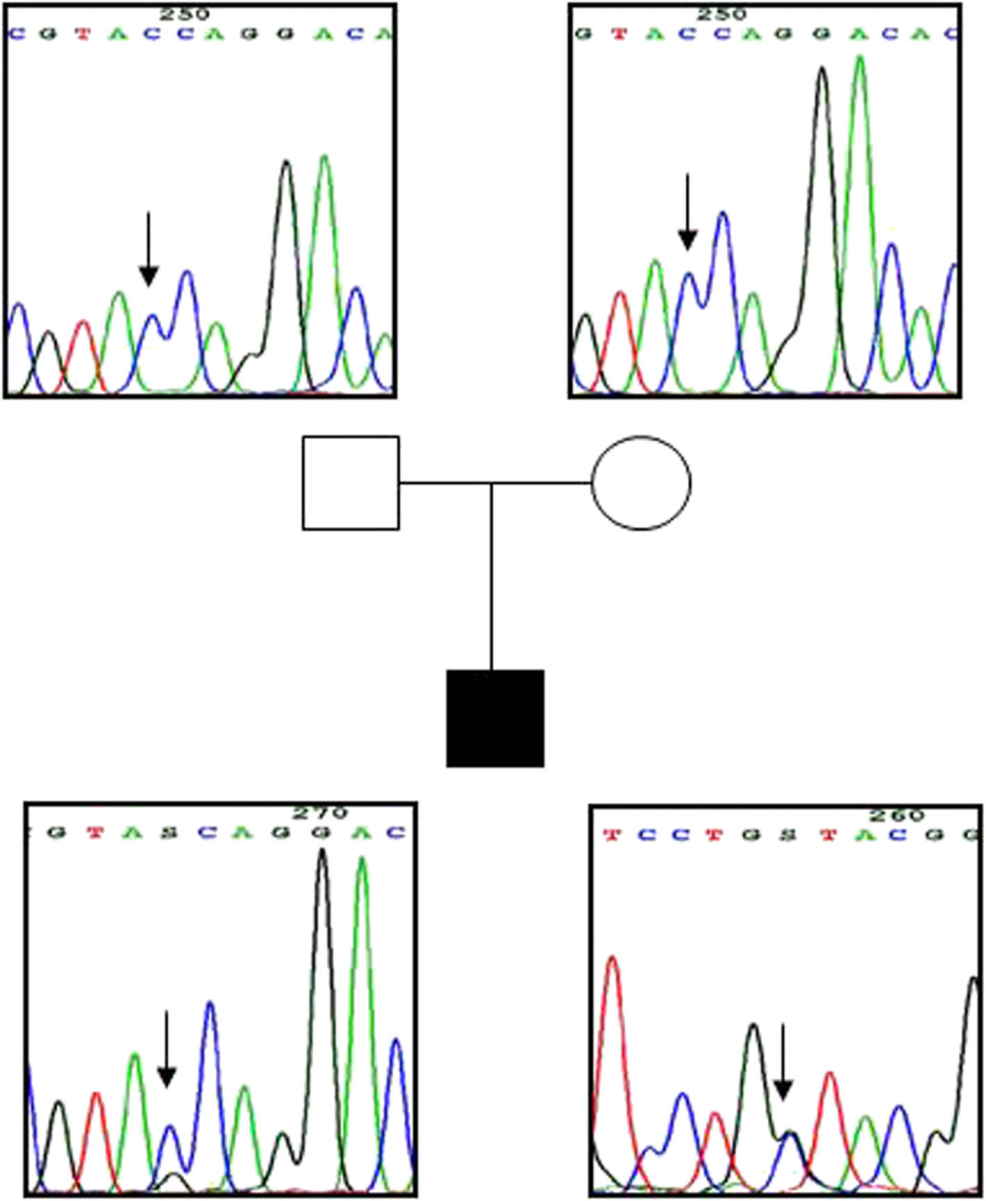
Chromatograms obtained from the genetic analysis of the patient and of his parents. Family tree with the corresponding chromatograms of *NKX2.1* exon 2 showing c.390C > G:p. (Tyr130Term) mutation (arrow): the father and mother are homozygous for the wild-type nucleotide (upper panels, sense strand), the proband is heterozygous for the mutation (bottom panels, sense and antisense strands)

The mutation was not detected in the parents, as shown in Fig. 1. Upon administration of the replacement therapy, the patient showed a progressive improvement in neuromotor and growth development. Of note, the height increased from −0,63 SDS to −0,12 SDS after one year of treatment.

## Discussion

We report the case of an infant with a novel mutation of the *NKX2.1* gene, showing neurological symptoms, with mild impairment of thyroid function, in the absence of neonatal respiratory distress, as a typical example of an already established group of disorders namely *NKX2.1*-related disorders.

The phenotypic spectrum of *NKX2.1*-related disorders is highly variable and ranges from abnormalities in a single organ system to any combination of brain, thyroid and lung involvement. The neurological presentation may be the most frequent one. In particular BHC, the hallmark of *NKX2.1*-related disorders, is a classic early finding, as suggested by previous evidence [5], and is characterized by involuntary, irregular, jerk-like and continuous movements, frequently associated with hypotonia, ataxia and choreoathetosis. In some cases BHC is associated with respiratory distress syndrome or congenital hypothyroidism.

The case that we report here is characterized by mild clinical presentation. The movement disorders of the child were monitored starting from the first months of life and choreathetosis was evident at 8 months. Neonatal CH screening showed near-normal results. A mild form of hypothyroidism was detected in his first months of life and was responsive to a low dose of L-Thyroxine. No respiratory distress at birth and no respiratory symptoms during childhood were observed.

A novel *NKX2.1* nonsense mutation (Y130X) and a single base exchange in intron 2(c. 463 + 41C > T), not reported in the SNP database (NCBI), were identified. The mutations were not detected in parents, despite the known history of walking delay in the paternal line. This evidence indicates that we identified *de novo* mutations.

The phenotype observed in our patient was mild despite the presence of *NKX2.1* gene null mutation (Y130X). This discrepancy is not surprising in light of previous data showing the lack of genotype-phenotype correlation in *NKX2.1*-related disorders [7, 11, 18, 19], likely due to the pathogenetic role of genetic and environmental factors that need to be further investigated [20].

The pathogenetic mechanism of the mutation, as other truncating mutations, might involve haploinsufficiency [2, 8, 10, 12, 21–23].

However, we cannot exclude the potential activation of an alternative initiation site leading to a shorter product of 268 aminoacids with intact homeobox domain (HD) and TD and lacking the protein N-terminal. This product may partially preserve the protein function and therefore explain the mild phenotype of the patient.

Several nonsense *NKX2.1* mutations upstream to the DNA binding domain, which associate with a high phenotypic variability, have been previously reported [2, 7, 22, 24–27].

*Asmus et al.* [7], *Peall et al.* [22] and *Hamvas et al.* [24] reported three cases characterized by the same Y144X nonsense mutation and different phenotypes. *Asmus et al.* and *Peall et al.* reported the case of a patient with chorea, as the only clinical presentation. *Hamvas et al.* described a patient with hypotonia, development delay and recurrent pulmonary infections, who died of a respiratory sincytial virus infection.

Other nonsense *NKX2.1* mutations upstream to the DNA binding domain occuring with choreoathetosis, CH and respiratory distress have been reported by *Krude et al*. (C117X) [2], *Peall et al.* (W143X) [22], *Ferrara et al.* (S175X) [25] and *Nakamura et al.* (Y98X) [26].

The G142X nonsense mutation upstream to the *NKX2.1* DNA binding domain was described by *Salerno et al*. [27] in a patient with CH and respiratory distress but not chorea, whose father was a immune carrier.

Nevertheless, the high phenotypic variability, even in cases carrying the same mutation, is not restricted to *NKX2.1* nonsense mutations, but is a feature of all types of *NKX2.1* mutations.

Therefore, the hypothesis of the activation of an alternative initiation site with intact HD in the novel Y130X nonsense mutation, upstream to the *NKX2.1* DNA binding domain, seems to be remote. Functional studies are needed to definitively rule out this hypothesis.

## Conclusions

Previously published data do not allow a clear interpretation of the pathogenetic role of *NKX2.1* mutations in *NKX2.1*-related disorders, since a number of cases were not fully characterized for the phenotypic presentation of choreoathetosis. Moreover, the same mutation of the *NKX2.1* gene can present with diverse phenotypes and highly variable clinical manifestations. Our analysis suggests that in the absence of severe thyroid deficit or respiratory distress, neurological evaluation should guide the patient's management. The mild hypothyroidism, frequently observed in these cases, may not be detected by neonatal screening. The low frequency of CH cases due to *NKX2.1* mutations that are reported in literature is expected to be higher in a subgroup of patients selected according to the neurological presentation. In these patients, the analysis of *NKX2.1* mutational status is recommended.

## Consent

Written informed consent was obtained from the patient’s parents for the publication of this case report and any accompanying images. A copy of the written consent is available for the review by the Editor-in-Chief of this journal.

## Abbreviations

CH: Congenital hypothyroidism
BHC: Benign hereditary chorea
HD: Homeobox domain
